# Molecular Characteristics of Cisplatin-Induced Ototoxicity and Therapeutic Interventions

**DOI:** 10.3390/ijms242216545

**Published:** 2023-11-20

**Authors:** Winston J. T. Tan, Srdjan M. Vlajkovic

**Affiliations:** 1 Department of Physiology, Faculty of Medical and Health Sciences, The University of Auckland, Auckland 1023, New Zealand; winston.tan@auckland.ac.nz; 2 Eisdell Moore Centre, Faculty of Medical and Health Sciences, The University of Auckland, Auckland 1023, New Zealand

**Keywords:** cisplatin, ototoxicity, hearing loss, cochlea, oxidative stress, inflammation

## Abstract

Cisplatin is a commonly used chemotherapeutic agent with proven efficacy in treating various malignancies, including testicular, ovarian, cervical, breast, bladder, head and neck, and lung cancer. Cisplatin is also used to treat tumors in children, such as neuroblastoma, osteosarcoma, and hepatoblastoma. However, its clinical use is limited by severe side effects, including ototoxicity, nephrotoxicity, neurotoxicity, hepatotoxicity, gastrointestinal toxicity, and retinal toxicity. Cisplatin-induced ototoxicity manifests as irreversible, bilateral, high-frequency sensorineural hearing loss in 40–60% of adults and in up to 60% of children. Hearing loss can lead to social isolation, depression, and cognitive decline in adults, and speech and language developmental delays in children. Cisplatin causes hair cell death by forming DNA adducts, mitochondrial dysfunction, oxidative stress, and inflammation, culminating in programmed cell death by apoptosis, necroptosis, pyroptosis, or ferroptosis. Contemporary medical interventions for cisplatin ototoxicity are limited to prosthetic devices, such as hearing aids, but these have significant limitations because the cochlea remains damaged. Recently, the U.S. Food and Drug Administration (FDA) approved the first therapy, sodium thiosulfate, to prevent cisplatin-induced hearing loss in pediatric patients with localized, non-metastatic solid tumors. Other pharmacological treatments for cisplatin ototoxicity are in various stages of preclinical and clinical development. This narrative review aims to highlight the molecular mechanisms involved in cisplatin-induced ototoxicity, focusing on cochlear inflammation, and shed light on potential antioxidant and anti-inflammatory therapeutic interventions to prevent or mitigate the ototoxic effects of cisplatin. We conducted a comprehensive literature search (Google Scholar, PubMed) focusing on publications in the last five years.

## 1. Introduction

Platinum-based chemotherapeutic drugs, such as cisplatin, carboplatin, and oxaliplatin, are widely used in the treatment of solid malignant tumors in both adult and pediatric patients, including testicular, ovarian, cervical, breast, bladder, head and neck, and lung cancer. Cisplatin (cis-diamminedichloroplatinum (II)) is the most widely used platinum-based drug. Since its approval by the U.S. Food and Drug Administration (FDA) in 1978, cisplatin has been a core therapy in oncology and an essential systemic therapy for germ cell malignancies [1].

Cisplatin induces the crosslinking of DNA and the formation of DNA adducts, which ultimately trigger apoptosis in cancer cells [2,3]. Despite its clinical effectiveness, cisplatin can cause severe side effects due to its non-specific mechanisms of action, targeting both cancer cells and healthy tissues. The adverse effects of cisplatin are dose-dependent and include ototoxicity, nephrotoxicity, neurotoxicity, hepatotoxicity, gastrointestinal toxicity, and retinal toxicity. Ototoxicity is the most common side effect due to the accumulation and prolonged retention of cisplatin within cochlear tissues [4].

**Figure 1:**
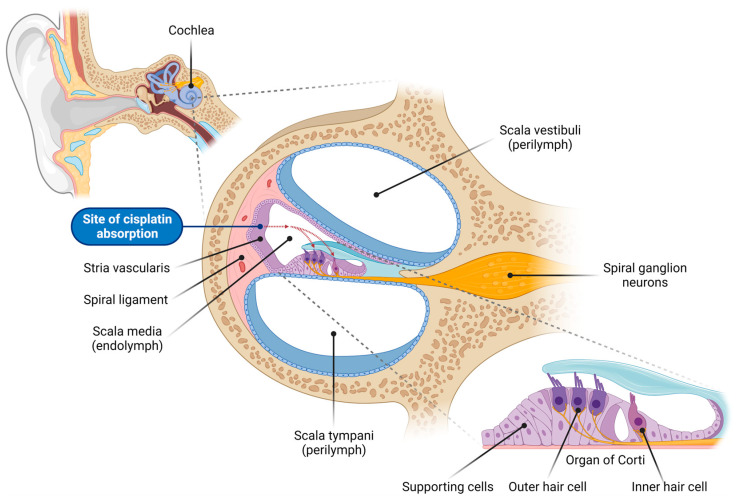
**Overview of the cochlear structure and the site of cisplatin absorption.** This illustration depicts the anatomical structure of the cochlea, the organ of hearing located within the inner ear, highlighting the various cell types vulnerable to cisplatin-induced damage. The cochlea consists of three fluid-filled compartments: scala vestibuli and scala tympani, filled with Na^+^-rich perilymph, and scala media, filled with K^+^-rich endolymph. The scala media houses the organ of Corti, which comprises the inner hair cells (responsible for auditory transduction), outer hair cells (critical for cochlear amplification), and surrounding supporting cells. Adjacent to the organ of Corti sits the lateral wall, comprising the stria vascularis (responsible for generating and maintaining the endocochlear potential, the driving force for sensory transduction) and spiral ligament (supporting the stria vascularis and cochlear fluid homeostasis). Spiral ganglion neurons innervate the sensory hair cells and transmit auditory signals to the auditory nuclei in the brainstem. Cisplatin enters the endolymph in the scala media through capillaries in the stria vascularis, and is subsequently absorbed by the sensory hair cells in the organ of Corti (red dashed arrows). This figure was created using BioRender.com (accessed on 16 November 2023).

Cisplatin ototoxicity mainly arises from effects on the sensory hair cells, spiral ganglion neurons (SGNs), and secretory and connective tissues (stria vascularis and spiral ligament) of the cochlea (Figure 1) [2]. Due to the absence of regenerative capacity in mammalian hair cells and SGNs, their death is irreversible, resulting in permanent sensorineural hearing loss. Cisplatin-induced ototoxicity is characterized by bilateral, moderate-to-profound high-frequency hearing loss, and a significant loss of outer hair cells (OHCs) in the basal turn of the cochlea. Although cisplatin primarily affects auditory function, inner ear toxicity can also present as tinnitus and infrequently as ear pain or balance disorder due to vestibulotoxicity [5,6]. Ototoxicity is determined by several factors, including the patient’s age, cumulative cisplatin dose, and predisposition [7]. Cisplatin-induced hearing loss affects 40–60% of adults, of which 18% have severe to profound hearing loss after cisplatin treatment, whilst tinnitus is present in 40% of cases [8]. Up to 60% of children treated with cisplatin are also affected by hearing loss [9]. Pediatric patients may experience speech and language development difficulties due to hearing impairments. Other consequences of cisplatin-induced hearing loss include social isolation, anxiety, and depression, negatively affecting overall well-being.

In addition to lowering the cisplatin dose or transitioning to a non-cisplatin treatment, both of which reduce the efficacy of chemotherapy, interventions that can prevent or restore hearing loss induced by cisplatin chemotherapy are lacking. Currently, managing cisplatin-induced hearing loss mainly involves the use of hearing aids. Recently, the antioxidant sodium thiosulfate (Pedmark) was approved by the U.S. FDA to prevent cisplatin-induced hearing loss in pediatric patients; however, its efficacy remains limited as 28–33% of the patients still suffered hearing loss [10,11,12,13]. Thus, there is still an unmet clinical need for interventions to manage cisplatin ototoxicity. Several promising preventative and therapeutic approaches are currently in various stages of preclinical or clinical development.

In summary, while platinum-based chemotherapeutic drugs are valuable in cancer treatment, their side effects, such as ototoxicity, necessitate the development of novel therapies to prevent hearing loss in oncological patients. This review explores the relationship between cisplatin, oxidative stress, and cochlear inflammation, shedding light on potential therapeutic interventions to mitigate ototoxicity.

## 2. Methods

For this narrative review, we conducted a comprehensive search of the literature utilizing Google Scholar and PubMed. The following search terms were employed: cisplatin AND ototoxicity; cisplatin AND hearing loss; cisplatin AND cochlea AND oxidative stress; cisplatin AND cochlea AND inflammation; cisplatin AND ototoxicity AND treatment. The search results were then examined according to their relevance to this review. We included only English-language publications, encompassing both preclinical (in vivo and in vitro) and clinical studies. Our selection was primarily focused on papers published within the last five years (2018 to 2023) to ensure an up-to-date review of the literature. In total, 146 papers were selected and referenced.

## 3. Cisplatin Uptake into the Cochlea

Cisplatin is a small, highly reactive molecule with no net charge that can passively diffuse across biological membranes down its electrochemical gradient at a rate correlated with lipophilicity [14]. The absorption of cisplatin does not saturate with increasing concentrations; therefore, the amount transported is proportionate to the amount administered [15].

**Figure 2:**
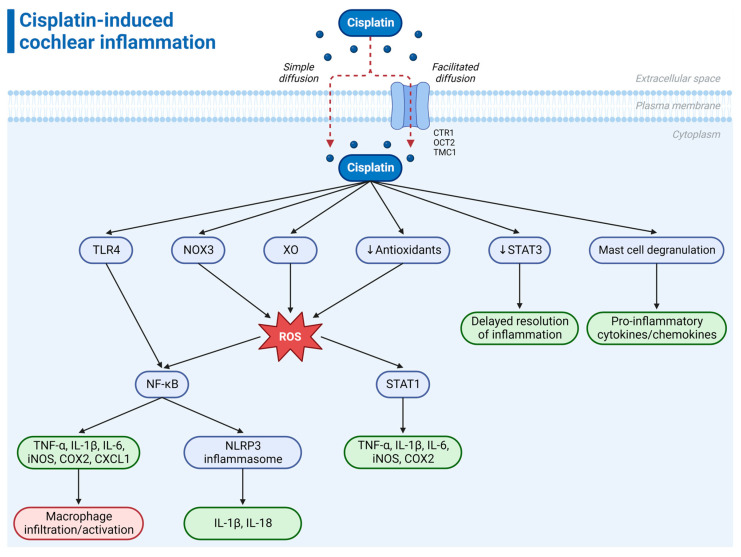
**Proposed mechanism of cisplatin-induced cochlear inflammation.** Following its entry into the cochlea and uptake by cells via either simple or facilitated diffusion, cisplatin induces an inflammatory response through various signaling pathways, including TLR4–NF-κB activation, TLR4–NF-κB–NLRP3 inflammasome activation, NOX3–NF-κB activation, NOX3–STAT1 activation, XO–NF-κB/STAT1 activation, reductions in antioxidant enzyme levels, STAT3 inhibition, and mast cell degranulation. The upregulation of pro-inflammatory mediators in the cochlea leads to the infiltration and activation of macrophages. Abbreviations: COX-2, cyclooxygenase 2; CTR1, copper-like transporter-1; CXCL1, chemokine (C-X-C motif) ligand 1; IL-1β, interleukin-1β; IL-18, interleukin-18; IL-6, interleukin-6; iNOS, inducible nitric oxide synthase; NF-κB, nuclear factor kappa B; NLRP3, NOD-like receptor protein 3; NOX3, NADPH oxidase 3; OCT2, organic cation transporter-2; ROS, reactive oxygen species; STAT1, signal transducer and activator of transcription-1; STAT3, signal transducer and activator of transcription-3; TNF-α, tumor necrosis factor-alpha; TLR4, Toll-like receptor 4; TMC1, transmembrane channel-like protein 1; XO, xanthine oxidase. This figure was created using BioRender.com (accessed on 16 November 2023).

The primary route of entry of cisplatin into the cochlea is via the vasculature in the stria vascularis (Figure 1) [4,16,17,18]. Following entry into the endolymph in the scala media, cisplatin is absorbed by the sensory hair cells across their apical membrane [19]. It has been proposed that simple diffusion accounts for approximately 50% of the initial uptake rate, and facilitated diffusion explains the remaining 50% [20]. Facilitated diffusion is mediated by various transduction channels and transporters in the plasma membrane (Figure 2). This includes transmembrane channel-like protein 1 (TMC1), which forms the mechanoelectrical transduction (MET) channel located at the tips of hair cell stereocilia, copper-like transporter-1 (CTR1), and organic cation transporter-2 (OCT2) [19,21,22].

CTR1 is highly expressed in cochlear tissues affected by cisplatin-induced ototoxicity, including the OHCs, stria vascularis, and SGNs [23]. Cisplatin accumulates 1.9-fold faster in immortalized human embryonic kidney cells (HEK 293) containing the full-length human *Ctr1* gene compared with control HEK 293 cells lacking the *Ctr1* gene, suggesting the critical role of CTR1 in cisplatin uptake [23].

Cisplatin can also enter supporting cells through non-selective transporters such as OCT2 [24] and induce toxicity by damaging their DNA and mitochondria. It has been postulated that apoptotic signals could propagate through gap junction channels in supporting cells, causing widespread cell death in the sensory epithelium [24].

Once taken up by the cell, cisplatin undergoes an aquation reaction, creating an activated form that can bind to and damage negatively charged macromolecules within the cell, including DNA, RNA, proteins, and lipids, leading to apoptotic cell death [25]. The cochlea has the capacity to retain cisplatin for an extended period compared with other organs [4]. In most organs (i.e., the kidney, lung, and heart), cisplatin is detected within one hour of injection and eliminated over days to weeks. In contrast, the mouse and human cochlea retain cisplatin for months to years following cisplatin treatment, with a high accumulation of cisplatin in the stria vascularis relative to other cochlear regions [4]. This prolonged retention of cisplatin within the cochlea likely contributes to its ototoxicity.

A recent study [26] revealed that cisplatin localizes to stress granules (SGs). These are non-membranous, irregularly shaped cellular compartments that are transiently assembled in response to various stress stimuli, playing a crucial role in cell survival [27,28]. SGs are composed of mRNA, RNA-binding proteins, translation factors, and other proteins, and are rapidly disintegrated and cleared upon removal of the stressor [27,28,29]. Martin et al. [26] demonstrated that SGs retain cisplatin for at least 24 h, leading to impairments in their assembly, including the reduced sequestration of DEAD-Box Helicase 3 X-Linked (DDX3X) signaling protein essential for inflammasome formation. This leads to alterations in SG dynamics and composition. Consequently, cisplatin-treated cells are unable to mount an SG response to additional stress, leaving cochlear cells vulnerable to future insults. The impaired SG response induced by cisplatin could potentially contribute to the development of cisplatin-induced ototoxicity.

## 4. Cisplatin-Induced DNA Damage

Cisplatin causes inter- and intra-strand DNA crosslinks [30]. Genomic DNA, particularly the N7 position of the guanine purine base, is the main target for cisplatin [30]. By coordinating with the N7 position of adjacent purine, this point of attack creates monofunctional adducts, providing an intra-strand DNA crosslink [31]. This crosslink results in a 36° bend and structural deformation of the DNA duplex, preventing the DNA from winding and suppressing DNA transcription [30]. The mismatch repair protein human MutS homolog 2 (hMSH2) and the nonhistone chromosomal high-mobility groups 1 and 2 (HMG1 and 2) are among the 20 proteins that recognize this DNA damage [32]. These proteins subsequently relay DNA damage signals to downstream signaling cascades, ultimately inducing apoptosis [32].

## 5. Oxidative Stress in Cisplatin-Induced Ototoxicity

Oxidative stress is a key contributor to the complex pathways responsible for cisplatin-induced cochlear injury and hearing loss. Extensive evidence implicates the increased production of reactive oxygen species (ROS), which triggers mitochondrial-mediated apoptosis in cochlear hair cells [7,19,21]. ROS are derivatives of molecular oxygen that occur as natural by-products of oxidative phosphorylation in mitochondria. Although ROS play a crucial role in cellular signaling in a variety of physiological processes, an intricate system of endogenous antioxidant enzymes, including superoxide dismutase (SOD), catalase (CAT), and glutathione (GSH), regulates ROS production and metabolism. An imbalance between ROS production and the level of antioxidants leads to oxidative stress, which can cause irreversible ROS-mediated damage to cellular DNA, proteins, and lipids [33]. In cochlear tissues with high metabolic demands, ROS-induced oxidative stress impacts mitochondrial DNA (mtDNA), respiratory chain proteins, and mitochondrial membranes, resulting in mtDNA mutations, protein oxidation, and lipid peroxidation, respectively [33,34,35,36,37]. This damage leads to mitochondrial dysfunction, which can facilitate further ROS production in a positive feedback loop.

Cisplatin increases the production of ROS in the cochlea primarily by activating NADPH oxidase-3 isoform (NOX3), a superoxide-generating enzyme highly expressed in the inner ear [38,39,40]. Cisplatin increases the expression of NOX3 in the supporting cells and OHCs, particularly in the basal turn of the cochlea [39]. The upregulation of NOX3 correlates with cisplatin-induced cochlear damage and hearing loss [39]. In contrast, suppression of NOX3 by gene knockout or short interfering RNA (siRNA) protects against cisplatin-induced ototoxicity [39,41].

Another superoxide-producing enzyme, xanthine oxidase (XO), has also been implicated as a source of cisplatin-induced ROS generation in the cochlea [38,42]. XO and its natural substrate, hypoxanthine, increase the intracellular calcium concentration in OHCs, which can modulate OHC electromotility [43]. Both ROS-producing enzymes (NOX3 and XO) set off a cascade of events involving apoptotic signaling pathways, ultimately leading to apoptosis and functional loss in the cochlea.

Cisplatin’s impact on antioxidant enzyme levels further exacerbates its ototoxic effects, as it directly reduces the levels of antioxidants, such as SOD, CAT, glutathione reductase (GR), and glutathione peroxidase (GSH-Px) in the cochlea [44,45]. ROS overproduction and the depletion of endogenous antioxidants in the cochlea ultimately lead to lipid peroxidation, as evidenced by elevated levels of malondialdehyde and 4-hydroxynonenal [44,45,46].

Cisplatin also contributes to increased nitric oxide (NO) production in the cochlea by upregulating inducible nitric oxide synthase (iNOS) levels. NO causes the nitration of cochlear proteins, severely disrupting their normal function [47,48,49]. NO reacts with NOX3-generated superoxide to form the highly reactive oxidant peroxynitrite, which reacts with proteins to form nitrotyrosine, a common marker of oxidative damage [48]. Cochlear protein nitration correlates with the dose-dependent increase in cisplatin-induced cochlear damage, indicating the pivotal role of protein nitration in cisplatin ototoxicity and hearing loss [48]. Cisplatin-induced nitrative stress in the cochlea primarily induces the nitration of a specific protein, LIM Domain Only 4 (LMO4), a transcriptional regulator that controls the choice between cell survival and death in OHCs, SGNs, and strial cells [48,50]. Nitration reduces the cochlear expression of LMO4, ultimately contributing to cisplatin-induced cochlear apoptosis and ototoxicity [48]. This was corroborated in LMO4 conditional knockout mice, which displayed enhanced susceptibility to cisplatin-induced apoptosis and hearing loss [50].

Recent evidence suggests that ferroptosis, a newly discovered non-apoptotic form of programmed cell death characterized by an iron-dependent accumulation of lipid peroxides and reduced mitochondrial membrane potential, also plays a role in cisplatin-induced ototoxicity [51,52]. The inhibition of ferroptosis with ferrostatin-1, a ferroptosis inhibitor, was shown to markedly attenuate cisplatin-induced hair cell damage by inactivating lipid peroxide radicals and preserving mitochondrial function [51,52].

Other evidence suggests that cisplatin causes a dysregulation of mitochondrial calcium homeostasis [53]. A recent study demonstrated that cisplatin acutely disrupts mitochondrial bioenergetics within the hair cells of the zebrafish lateral line by increasing mitochondrial activity and calcium levels [54]. The zebrafish lateral line is a valuable model for studying the roles of mitochondria in sensory hair cell pathologies and developing therapeutic strategies to prevent sensorineural hearing loss in humans. These alterations in mitochondrial function are also associated with elevated ROS levels and the activation of caspase-3-mediated apoptosis, indicating that mitochondrial dysfunction is an early event in the development of cisplatin-induced ototoxicity.

## 6. Blood–Labyrinth Barrier and Cochlear Inflammation

The blood–labyrinth barrier (BLB) separates the inner ear vasculature from the cochlear fluids, limiting the passage of blood-borne substances, similar to the blood–brain barrier [55,56]. It preserves inner ear fluid dynamics by restricting the permeability of vascular interfaces in the cochlea to potential infections and foreign antigens spreading via systemic circulation [55,56].

The BLB is characterized by non-fenestrated capillaries, where vascular endothelial cells are connected by tight junctions [55,56]. The BLB also comprises perivascular macrophage-like melanocytes (PVM/Ms) located between the marginal and basal cells of the stria vascularis. These cells are associated with microvessels through their cytoplasmic processes and wrap around endothelial cells and pericytes, reinforcing the BLB [57,58,59]. The tight junctions, pericytes, and PVM/Ms play critical roles in regulating the integrity of the BLB for maintaining inner ear homeostasis [59].

Despite clear evidence of the BLB, various insults can induce inflammatory responses in the cochlea, including noise exposure, ototoxic drugs, surgical stress, and mitochondrial damage [58,60,61,62,63]. Inflammatory responses in the cochlea involve the recruitment and tissue infiltration of circulating monocytes and the upregulation and release of various pro-inflammatory mediators (cytokines, chemokines, and cell adhesion molecules). 

Under steady-state conditions, the cochlea contains a distinct resident population of bone-marrow-derived macrophages, predominantly localized in the spiral ligament, spiral limbus, and scala tympani [57,58]. Cochlear macrophages differ in their phenotypic and functional states and are uniformly distributed along the cochlear length [64]. The spiral ligament is the primary site of cochlear inflammation, where resident cochlear macrophages communicate with spiral ligament fibrocytes and play a vital role in mediating the inflammatory response [65]. Upon activation, the resident cochlear immune cells and infiltrated macrophages secrete various pro-inflammatory and anti-inflammatory mediators involved in the initiation, maintenance, and resolution phase of the cochlear inflammatory response. It has been postulated that cochlear inflammation plays a significant role in the pathogenesis of cisplatin- and aminoglycoside-induced ototoxicity [66,67,68], but is also implicated in noise-induced and age-related hearing loss [60,61,63,69,70,71,72,73]. However, the exact roles of these resident and infiltrated immune cells in resolving cochlear injury are still not fully understood.

## 7. Cisplatin-Induced Cochlear Inflammation

Cisplatin causes structural and functional damage to the stria vascularis and can compromise the integrity and permeability of the BLB [74]. It is well-documented that cisplatin induces ultrastructural alterations in the marginal cells, endothelial cells, and pericytes and significantly reduces the endocochlear potential, the driving force for sensory transduction [75,76,77]. Cisplatin also reduces the expression of the gap junction proteins, connexin 26 and connexin 43, in the marginal and basal cells and induces the activation of PVM/Ms and their secretion of interleukin-1β (IL-1β) [76].

The hyperpermeability of the BLB is caused by cisplatin-induced changes to the number and morphology of PVM/Ms and pericytes and the activation of hypoxia-inducible factor 1 alpha (HIF-1α) and the downstream target vascular endothelial growth factor (VEGF) [74]. This increased permeability of the BLB results in the accumulation of cisplatin in the stria vascularis and allows inflammatory cells (macrophages) to enter the cochlea. Previous studies have alluded to the possibility of systemic inflammation increasing the cochlear uptake of certain ototoxic medications across the BLB, which can further exacerbate cochlear inflammation and tissue damage [78].

While DNA damage and oxidative stress have been recognized as central mechanisms underlying cisplatin-induced ototoxicity, the pro-inflammatory nature of cisplatin suggests that its ototoxicity could be closely related to inflammation. Cisplatin triggers a cascade of inflammatory responses within the cochlea, marked by the release of pro-inflammatory cytokines, such as tumor necrosis factor-alpha (TNF-α), IL-1β, and interleukin-6 (IL-6) (Figure 2). The amount of pro-inflammatory cytokines in the cochlea is directly proportional to the cisplatin dosage and drug exposure time [79]. The excessive release of inflammatory mediators in the inner ear may lead to cell death, cochlear damage, and, eventually, hearing loss. Inflammation in the inner ear caused by exposure to ototoxic drugs is not a response to a pathogen; thus, it has been referred to as sterile inflammation [80].

Cisplatin induces the expression of pro-inflammatory cytokines through the activation of the transcription factor nuclear factor kappa B (NF-κB) [81,82]. Due to its role in controlling innate and adaptive immune responses, NF-κB is regarded as a master regulator of inflammation [83,84]. NF-κB is activated by Toll-like receptor 4 (TLR4), which belongs to the pattern recognition receptor (PRR) family [85,86]. TLR4 connects extracellular signals with intracellular signaling pathways, mediating inflammation in the inner ear [79,85]. Cisplatin binding to TLR4 activates a series of signaling events mediated by cytokines and chemokines that can direct the development of inflammation through self-amplifying signaling cascades [79,85]. Cytokines and chemokines elicit immune cell activation and the recruitment of inflammatory cells (macrophages) to the cochlea, thereby exacerbating cisplatin-induced cochlear damage [68]. Furthermore, NOX3 and ROS can also activate NF-κB, which, in turn, activates caspases 3 and 9 involved in cell apoptosis [87]. NF-κB also increases the expression of iNOS, which enhances NO production [47]. Sustained inflammation eventually leads to apoptosis of cochlear cells, particularly the OHCs, resulting in hearing loss.

Other studies have shown that cisplatin stimulates the activation of the NOD-like receptor protein 3 (NLRP3) inflammasome [88,89], a cytosolic multi-protein complex that plays a pivotal role in regulating the innate immune and inflammatory responses [90,91]. Activated NLRP3 promotes the cleavage of protease caspase-1, facilitating the production and secretion of pro-inflammatory cytokines, including IL-1β and IL-18 [90,91]. NLRP3 inflammasome activation may underlie cisplatin-induced inflammatory programmed cell death (pyroptosis) in the stria vascularis marginal cells [92]. It has been suggested that thioredoxin-interacting protein (TXNIP) is a potential upstream signal that activates the NLRP3 inflammasome [92]. Furthermore, the NLRP3 inflammasome is likely activated through the TLR4 signaling pathway via NF-κB [88]. The TLR4/NF-κB/NLRP3 signaling pathway may thus play a crucial role in cisplatin-induced cochlear inflammation.

Cisplatin enhances the expression of signal transducer and activator of transcription-1 (STAT1) whilst downregulating the expression of STAT3 in the cochlea [93,94,95,96]. STAT1 promotes the expression of multiple pro-inflammatory mediators, including cyclooxygenase 2 (COX-2), iNOS, TNF-α, IL-1β, and IL-6, further exacerbating cochlear inflammation. ROS activates STAT1 through NOX3 NADPH oxidase [96]. Conversely, STAT3 is a pleiotropic prosurvival transcription factor that inhibits apoptosis and aids in the resolution of inflammation. Consequently, its downregulation renders cells susceptible to oxidative stress and inflammation-induced damage. This interplay between STAT1 and STAT3 creates a positive feedback loop, prolonging the cochlear inflammatory response induced by cisplatin and delaying the resolution phase of inflammation [93,94,95,96]. STAT1 and STAT3 thus present promising targets for therapeutic interventions to mitigate cisplatin-induced ototoxicity. The inhibition of STAT1 may suppress inflammation and apoptosis, while enhancing STAT3 expression may promote cell survival (see Section 8). Another member of the STAT family, STAT6, is also involved in the cisplatin-induced production of pro-inflammatory cytokines in the cochlea and may also serve as a potential therapeutic target for cisplatin ototoxicity [97].

Chemokines, a group of secreted proteins within the cytokine family whose generic function is to induce cell migration, have been implicated in the initiation of the cochlear inflammatory response induced by cisplatin. Released by resident macrophages and supporting cells, chemokines aid in the recruitment and infiltration of immune cells from the circulation to the site of tissue injury. A recent study [98] suggested that chemokine (C-X-C motif) ligand 1 (CXCL1) acts as an early player in cisplatin-induced ototoxicity, as increased levels of CXCL1 were detected in the serum and cochlea 24 h after cisplatin administration. Al Aameri et al. [98] demonstrated a time-dependent increase in CXCL1 expression in the SGNs and the organ of Corti following cisplatin treatment. This was associated with a progressive increase in the number of CD45, CD68, and Iba-1-positive immune cells (macrophages) in the cochlea. Inhibition of CXCR2, the receptor for CXCL1, by the intratympanic administration of SB225002, a selective CXCR2 antagonist, reduced immune cell infiltration in the cochlea and protected against cisplatin-induced hair cell loss and hearing loss [98]. SB225002 also reduced the expression of various other pro-inflammatory mediators, including NOX3, iNOS, TNF-α, IL-6, and COX-2.

Mast cells, densely granulated cells of the myeloid lineage, have been identified in the modiolus, spiral limbus, and spiral ligament of the rodent cochlea [99]. The degranulation of mast cells results in the release of inflammatory mediators, including various cytokines and chemokines involved in cisplatin-induced cochlear damage [100]. Recent studies have shown that cisplatin alters the number and morphology of mast cells [99,100]. The inhibition of mast cell degranulation with cromoglicic acid (cromolyn) protects against the cisplatin-induced loss of sensorineural tissues [100].

In summary, cisplatin-induced cochlear inflammation involves the activation of TLR4 by cisplatin, leading to the production of pro-inflammatory mediators through NF-κB/NLRP3 signaling (Figure 2). The upregulation of STAT1 and downregulation of STAT3 by cisplatin further contribute to inflammation by inducing the expression of pro-inflammatory mediators and delaying the resolution of inflammation. Furthermore, the interaction between ROS and inflammatory mediators leads to a positive feedback loop, further exacerbating cochlear damage.

## 8. Antioxidant and Anti-Inflammatory Treatments for Cisplatin-Induced Ototoxicity

Several potential therapies have been explored in preclinical (animal models and cell lines) and clinical studies to prevent or attenuate cisplatin-induced ototoxicity by targeting oxidative stress and cochlear inflammation (Table 1) [101,102,103,104,105]. These approaches hold promise for the development of treatments to alleviate the ototoxic effects of cisplatin and prevent hearing loss. The main condition is that any treatment that prevents cisplatin ototoxicity should not interfere with its tumor-killing activity. Local intratympanic otoprotectant delivery has become an increasingly attractive proposition to prevent cisplatin-induced hearing loss compared with systemic administration. Local delivery bypasses the BLB and achieves higher drug concentrations in the cochlea without systemic side effects. Other innovations in drug delivery systems include nanoparticles, hydrogels, and environmental stimuli systems applied to the inner ear [7].

The antioxidant sodium thiosulfate (Pedmark, Fennec Pharmaceutical Inc., Research Triangle Park, NC, USA) recently became the first FDA-approved treatment for preventing cisplatin-induced hearing loss in pediatric patients aged 1 month and older with localized, non-metastatic solid tumors [10,13]. This approval followed two Phase 3 clinical trials (SIOPEL 6, ClinicalTrial.gov identifier: NCT00652132 and COG ACCL0431, ClinicalTrials.gov identifier: NCT00716976) where sodium thiosulfate (STS) was administered intravenously over 15 min, starting 6 h after cisplatin chemotherapy [11,12,106,107]. STS significantly reduced the incidence of hearing loss in children aged 1 month to 18 years by acting through two distinct mechanisms. Firstly, STS acts as an ROS scavenger and increases the levels of endogenous antioxidants after entering hair cells via sodium sulfate cotransporter 2 [13,108]. Secondly, it directly interacts with cisplatin, neutralizing its ototoxic effects [13,108]. The delayed administration of STS after cisplatin treatment enables cisplatin to exert its anti-cancer properties before being neutralized by STS [13,108,109].

*N*-acetyl cysteine (NAC) functions as both a direct free radical scavenger and a substrate for the synthesis of the antioxidant glutathione. Although it has demonstrated efficacy in preventing cisplatin-induced ototoxicity in animals when administered intratympanically [110], human studies have yielded mixed results with intratympanic NAC administration [111,112]. The optimal dosage and efficacy of intratympanic NAC injection in reducing cisplatin-induced hearing loss in head and neck cancer patients is being evaluated in a current Phase 2 clinical trial (ClinicalTrials.gov identifier: NCT04291209) [113]. Interestingly, a recent study [114] has suggested that mannitol, a diuretic that transiently increases the BLB permeability, can enhance the otoprotective effects of NAC and STS.

Ebselen, a synthetic mimic of the antioxidant enzyme GPx1 with anti-inflammatory properties, has also demonstrated beneficial effects against cisplatin ototoxicity [42,45,115,116,117]. Currently, SPI-1005, a proprietary oral formulation of ebselen, is undergoing Phase 2 clinical trials (ClinicalTrials.gov Identifier: NCT01451853) [118] to assess its potential in preventing and treating cisplatin-induced hearing loss and tinnitus. This clinical trial is aiming to evaluate the safety and efficacy of three different doses of SPI-1005 administered orally to patients diagnosed with head and neck or non-small cell lung cancer.

Another antioxidant that has shown promising results is D-methionine. This sulfur-containing amino acid acts as a direct scavenger of free radicals and protects the enzymatic activity of endogenous antioxidants. In animal studies, D-methionine demonstrated protective effects against cisplatin-induced ototoxicity through systemic [44,119,120,121] and local administration onto the round window membrane [122,123]. Campbell et al. [119] established that both oral and injected D-methionine yielded comparable levels of otoprotection, effectively preventing cisplatin-induced auditory brainstem response (ABR) threshold shifts. Similarly, a Phase 2 clinical trial involving cancer patients revealed that those who received oral D-methionine prior to each cisplatin dose showed reduced ABR threshold shifts compared with the placebo group [124].

Dexamethasone, a corticosteroid with anti-inflammatory properties, has shown some degree of protection against cisplatin ototoxicity in animal studies after intratympanic administration [125,126,127,128,129,130,131,132,133]. However, clinical studies have demonstrated limited otoprotective effects of intratympanic dexamethasone [134,135]. The most recent clinical trial (ClinicalTrials.gov identifier: NCT02997189) [136] investigating OTO-104, a sustained-release hydrogel formulation of dexamethasone, in preventing cisplatin-induced ototoxicity was terminated due to negative efficacy outcomes in a related clinical study.

Due to the pivotal role of the pro-inflammatory cytokine TNF-α in the development of cisplatin-induced ototoxicity, preclinical studies have demonstrated the otoprotective effect of the TNF-α inhibitor etanercept. Rats that received intratympanic etanercept prior to cisplatin administration exhibited significantly reduced ABR threshold shifts and increased OHC survival compared with non-treated controls [96]. Interestingly, etanercept injection also led to significant decreases in both serum and cochlear mRNA and protein levels of not only TNF-α, but also IL-1β and IL-6, suggesting that TNF-α contributes to the expression of other pro-inflammatory cytokines [81]. These findings demonstrate the potential of TNF-α inhibition as a therapeutic approach to preventing cisplatin-induced hearing loss [96].

Controlling inflammation by regulating STAT1 and STAT3-dependent pathways in the cochlea could serve as an effective therapeutic approach for cisplatin-induced ototoxicity, as evidenced by various preclinical studies [93,94,96,137,138]. The inhibition of STAT1 using short interfering RNA (siRNA) reduced the expression of pro-inflammatory mediators and cell apoptosis in the rat cochlea, protecting against ototoxicity [96]. Intratympanic administration of R-phenylisopropyladenosine R-PIA, an adenosine A_1_ receptor agonist, protected against OHC damage and hearing loss by reducing ROS production and STAT1-mediated inflammation [137,139]. Cisplatin ototoxicity in rats was also reduced by adenosine amine congener (ADAC), acting on adenosine A_1_ receptors [140]. The phytopharmaceutical aucubin, a member of the iridoid glycoside family that exhibits antioxidant and anti-inflammatory properties, protects against cisplatin-induced cochlear damage in vitro and in vivo by activating the STAT3 pathway [138]. Direct activation of the transient receptor potential vanilloid 1 (TRPV1) channel with capsaicin, a TRPV1 agonist extracted from chili peppers, increased the ratio of phosphorylation-activated STAT3/STAT1, leading to an anti-inflammatory response and protection from cisplatin ototoxicity [93]. Furthermore, apelin-13, a peptide hormone and endogenous ligand of the apelin receptor APJ, protected against cisplatin-induced ototoxicity and inhibited ROS production, apoptosis, and pro-inflammatory cytokine expression by reducing the phosphorylation and activation of STAT1 while increasing the phosphorylation and activation of STAT3 [94].

Other studies suggest that some polyphenols can modulate both oxidative stress and inflammation in the cochlea [141]. The polyphenol curcumin reduced cisplatin-induced hearing loss and suppressed NF-κB-related inflammation pathways in the cochlea while providing optimal chemosensitivity [141,142]. Recently, treatment with avenanthramide-C (AVN-C), a potent naturally occurring polyphenolic compound found exclusively in oats, reduced ROS production, enhanced the survival of OHCs and inner hair cell (IHC) presynaptic ribbons, and protected against cisplatin-induced hearing loss in mice [143]. When administered to a mouse auditory HEI-OC1 cell line prior to cisplatin exposure, AVN-C significantly reduced ROS production and mitigated cisplatin-induced inflammation by reducing the expression of the pro-inflammatory mediators, IL-6, IL-1β, TNF-α, iNOS, and COX2 [143]. Observations from previous studies suggest that AVN-C decreases the secretion of pro-inflammatory cytokines by inhibiting both NF-κB activity and mast cell degranulation [144,145]. These findings suggest AVN-C is a potential therapeutic candidate for ameliorating cisplatin-induced oxidative stress and cochlear inflammation.

Statins, drugs that lower cholesterol, have also shown antioxidant and anti-inflammatory effects and effectiveness against cisplatin ototoxicity in an animal model [146]. However, their mechanism of action is yet to be determined.

**Table 1:** Summary of antioxidant and anti-inflammatory treatments for cisplatin-induced ototoxicity in various stages of preclinical and clinical development. Abbreviations: IP, intraperitoneal; IT, intratympanic; IV, intravenous.

Therapeutic Agent	Mechanism of Action	Administration Route	Stage of Development
Sodium thiosulfate (Pedmark)	Antioxidant	IV	FDA-approved [11,12,106,107]
*N*-acetyl cysteine (NAC)	Antioxidant	IT	Preclinical [110] Clinical (completed) [111,112] Clinical (Phase 2, ongoing) [113]
Ebselen (SPI-1005)	Antioxidant Anti-inflammatory	Oral	Preclinical [42,45,115,116,117] Clinical (Phase 2, ongoing) [118]
D-methionine	Antioxidant	Oral, IP, local	Preclinical [44,119,120,121,122,123] Clinical (Phase 2, completed) [124]
Dexamethasone	Anti-inflammatory	IT, local	Preclinical [125,126,127,128,129,130,131,132,133] Clinical (completed) [134,135]
Etanercept	Anti-inflammatory	IT	Preclinical [81,96]
R-phenylisopropyladenosine (R-PIA)	Anti-inflammatory	IT	Preclinical [137]
Adenosine amine congener (ADAC)	Antioxidant	IP	Preclinical [140]
Aucubin	Antioxidant Anti-inflammatory	IT	Preclinical [138]
Capsaicin	Antioxidant Anti-inflammatory	Oral, IT	Preclinical [93]
Apelin-13	Antioxidant Anti-inflammatory	IP	Preclinical [94]
Curcumin	Antioxidant Anti-inflammatory	IP	Preclinical [142]
Avenanthramide-C (AVN-C)	Antioxidant Anti-inflammatory	IP	Preclinical [143]
Statins	Antioxidant Anti-inflammatory	Oral	Preclinical [146]
SB225002	Anti-inflammatory	IT	Preclinical [98]

## 9. Conclusions and Perspectives

Cisplatin-induced ototoxicity remains a significant concern in chemotherapy, impacting the quality of life of cancer survivors. Over the past decade, research has shed new light on the fundamental mechanisms underlying cisplatin-induced ototoxicity. An increasing body of evidence points towards complex interplays between oxidative stress and inflammation in the cochlea, culminating in cell death through the activation of mitochondrial apoptotic pathways.

These advances in our understanding of the mechanisms of cisplatin-induced ototoxicity have led to the discovery of several promising otoprotective therapies aimed at suppressing oxidative stress and inflammation in the cochlea. While many therapies have shown promise in preclinical studies, few have demonstrated otoprotective efficacy in clinical studies, including sodium thiosulfate, the only FDA-approved treatment thus far. Further research is needed to comprehensively evaluate the effectiveness and safety of other pharmacological interventions currently in clinical development.

A potential therapeutic strategy to combat sensorineural hearing loss associated with cisplatin could involve a multifaceted combination therapy called “cocktail therapy”, which simultaneously targets the primary mechanistic pathways through additive or synergistic effects. This approach would encompass compounds specifically directed at counteracting mitochondrial oxidative stress, reducing cochlear inflammation, and inhibiting mitochondrial apoptosis. Importantly, the recent success of sodium thiosulfate highlights the crucial need to identify the optimal onset and duration of treatment to effectively prevent sensorineural hearing loss while preserving the chemotherapeutic efficacy of cisplatin.

With the exciting announcement of the FDA approval of the first therapeutic to prevent cisplatin-induced ototoxicity and the numerous promising preclinical and clinical trials currently in progress, it is foreseeable that more clinically approved otoprotective therapies to address cisplatin-induced hearing loss will emerge in the future and improve treatment outcomes in cancer patients.

## Data Availability

Data sharing is not applicable.
